# Embedded axonal fiber tracts improve finite element model predictions of traumatic brain injury

**DOI:** 10.1007/s10237-019-01273-8

**Published:** 2019-12-06

**Authors:** Marzieh Hajiaghamemar, Taotao Wu, Matthew B. Panzer, Susan S. Margulies

**Affiliations:** 1 Wallace H. Coulter Department of Biomedical Engineering, Georgia Institute of Technology and Emory University, U.A. Whitaker Building, 313 Ferst Drive, Atlanta, GA 30332 USA; 2 Department of Mechanical and Aerospace Engineering, University of Virginia, 4040 Lewis and Clark Dr., Charlottesville, VA 22911 USA

**Keywords:** Multi-scale finite element modeling, Axonal injury prediction, Diffusion tensor imaging, Tractography, Axonal tract network

## Abstract

With the growing rate of traumatic brain injury (TBI), there is an increasing interest in validated tools to predict and prevent brain injuries. Finite element models (FEM) are valuable tools to estimate tissue responses, predict probability of TBI, and guide the development of safety equipment. In this study, we developed and validated an anisotropic pig brain multi-scale FEM by explicitly embedding the axonal tract structures and utilized the model to simulate experimental TBI in piglets undergoing dynamic head rotations. Binary logistic regression, survival analysis with Weibull distribution, and receiver operating characteristic curve analysis, coupled with repeated *k*-fold cross-validation technique, were used to examine 12 FEM-derived metrics related to axonal/brain tissue strain and strain rate for predicting the presence or absence of traumatic axonal injury (TAI). All 12 metrics performed well in predicting of TAI with prediction accuracy rate of 73–90%. The axonal-based metrics outperformed their rival brain tissue-based metrics in predicting TAI. The best predictors of TAI were maximum axonal strain times strain rate (MASxSR) and its corresponding optimal fraction-based metric (AF-MASxSR_7.5_) that represents the fraction of axonal fibers exceeding MASxSR of 7.5 s^−1^. The thresholds compare favorably with tissue tolerances found in in–vitro/in–vivo measurements in the literature. In addition, the damaged volume fractions (DVF) predicted using the axonal-based metrics, especially MASxSR (DVF = 0.05–4.5%), were closer to the actual DVF obtained from histopathology (AIV = 0.02–1.65%) in comparison with the DVF predicted using the brain-related metrics (DVF = 0.11–41.2%). The methods and the results from this study can be used to improve model prediction of TBI in humans.

## Introduction

Traumatic brain injury (TBI) represents a significant public health burden in the USA. In 2013 alone, there were approximately 2.8 million TBI-related emergency department (ED) visits, hospitalizations, and deaths occurred in the USA (Taylor et al. 2017). The rate of TBI has continued to increase, and it has been anticipated to become the major cause of death and disability by the year 2020, according to the World Health Organization (Hyder et al. 2007). TBI could lead to acute and long-term cognitive, behavioral, neurological, and possibly neurodegeneration impairments (Bazarian et al. 2009). However, definite detection of traumatic axonal injury (TAI), which is one of the most prevalent pathological features of TBI, remains a clinical challenge. TAI typically results from biomechanical events that induce rapid head movement which instigates dynamic brain tissue deformation and stretch of the axons. Stretching the axons beyond a critical threshold can lead to axonal swelling, which is one of the morphological hallmarks of TAI pathology, and eventually axonal transport impairment (Smith and Meaney 2000; Smith et al. 1999). In–vivo, in–vitro, and ex–vivo studies conducted on isolated nerve fibers or axons (Bain and Meaney 2000; Bain et al. 2001; Galbraith et al. 1993; Singh 2017; Singh et al. 2006, 2009), axonal or neuronal cultures (Bar-Kochba et al. 2016; Cullen et al. 2007; LaPlaca et al. 2005; Nakadate et al. 2017; Smith et al. 1999), and brain culture slices (Cater et al. 2006; Elkin and Morrison 2007; Morrison et al. 2000) have shown that several biomechanical parameters related to deformations of axons, nerve fibers or brain tissue such as shear or axial strain, strain tensor components, principal strains, and their rates correlate with the risk of injury. With the growing rate of TBI and their devastating outcomes, predictive tools are necessary to guide the development of preventative devices, improve the management/treatment outcomes, and reduce the risk of a second injury (Control and Prevention 2015).

Finite element models (FEM) are valuable tools for predicting TBI in the intact head. They can provide estimates of tissue responses such as strain, stress, and their rates during an event, using the kinematics experienced by the head as input. FEM can be used either to determine tissue injury thresholds or to predict the possible presence and degree of brain injury following a head trauma incident, or both (Coats et al. 2012; Colgan et al. 2010; Garimella et al. 2018; Giordano and Kleiven 2014a, b; Giordano et al. 2017; Hajiaghamemar et al. 2019; King et al. 2003; Sahoo et al. 2014, 2016; Sullivan et al. 2015; Wright et al. 2013; Wright and Ramesh 2012; Zhao et al. 2017). Tissue injury thresholds are determined by simulating a set of rapid head rotation and/or biomechanical head impact events, with known injury status. The presence and degree of brain injury are predicted by simulating the event and using pre-determined tissue injury threshold criteria. Either way, these studies correlate kinematics and tissue responses with resulting brain tissue injuries. In this communication, we improve the reliability of the outcomes of these finite element (FE) studies in three respects: (1) the biofidelity of the FEM, in terms of the anatomical details, material properties, and the validation process used to evaluate the tissue response; (2) the accuracy of kinematic inputs; and (3) the reliability of the process used to develop and evaluate the tissue injury metrics.

As background, the biofidelity of the head FEMs has been improved over the years in several ways. From the material modeling perspective, most of the previous FE studies modeled the brain tissue as an isotropic hyperelastic viscoelastic material (Chatelin et al. 2011; Coats et al. 2012; Colgan et al. 2010; Hajiaghamemar et al. 2019; King et al. 2003; Kleiven 2007; Maltese and Margulies 2016; Sullivan et al. 2015; Takhounts et al. 2003; Zhao et al. 2017). This assumption is acceptable for the gray matter which has an isotropic structure and material response. However, white matter from the corona radiata, corpus callosum and brainstem has been shown in many in–vitro studies (Arbogast and Margulies 1999; Feng et al. 2013; Ning et al. 2006; Prange and Margulies 2002) to consist of aligned axonal fiber bundles predominantly and has demonstrated a mechanically anisotropic material behavior. Anisotropic viscous hyperelastic constitutive models for brain material were developed (Chatelin et al. 2013; Cloots et al. 2012; Gasser et al. 2005; Wu et al. 2019) and implemented in the FEMs in more recent studies (Colgan et al. 2010; Ganpule et al. 2017; Giordano and Kleiven 2014a; Sahoo et al. 2014; Wright and Ramesh 2012). Moreover, with the advancements of the imaging techniques, the biofidelity of the FEMs has been, and continues to be, improved in terms of the brain geometry and incorporation of more anatomical details. Recently, some FE studies have incorporated the information of the axonal orientations into the FEMs utilizing diffusion tensor imaging (DTI) technique (Chatelin et al. 2011; Colgan et al. 2010; Sahoo et al. 2016; Sullivan et al. 2015; Wright and Ramesh 2012). In these studies, the brain tissue was modeled with an isotropic (Chatelin et al. 2011; Sullivan et al. 2015) or fiber-reinforced anisotropic (Giordano and Kleiven 2014b; Giordano et al. 2017; Sahoo et al. 2014; Wright and Ramesh 2012) material, and the brain biomechanical responses were projected onto the dominant axonal tract orientation at each brain element to calculate the axonal tract-oriented response. The incorporation of the axonal orientation information and the inclusion of anisotropy into the constitutive model of brain tissue have been shown to improve the biofidelity and the injury prediction performance of the FE brain models (Giordano et al. 2014; Wright and Ramesh 2012). More recently, a method was developed that can explicitly embed axonal tractography at a mesoscopic spatial resolution comparable to diffusion tensor imaging (DTI). The embedded element method, which has been recently introduced to TBI research community for human models, includes multiple fiber paths in each brain element and explicitly incorporates the axonal fiber structural network into the brain FE models using tractography data (Garimella and Kraft 2017; Garimella et al. 2018; Wu et al. 2019). In this paper, we improve the biofidelity of our pig FEM using this method, which to our knowledge has not been used in any animal TBI studies.

In this paper, we also pay attention to the accuracy of the kinematic data to enhance the credibility of FE results for the purpose of injury prediction. Most of the human FE studies have used kinematic inputs obtained from laboratory accident reconstructions of sports incidents documented with limited accuracy and precision (Giordano and Kleiven 2014b; Kleiven 2007; Sahoo et al. 2016; Zhao et al. 2017). For instance, in the laboratory reconstructed NFL head impact dataset, which is the most common dataset used in FE studies, there was up to 25% error reported in the kinematic data (Newman et al. 2005; Pellman et al. 2003; Sanchez et al. 2019). In contrast, in this paper we use animal TBI studies, in which experimental head kinematic data are recorded in a controlled laboratory setting, and the actual location and extent of axonal injury are available after killing. Thus, the coupling of computational and experimental models of animals enable the study of tissue deformations that lead to actual tissue injury, and thresholds can be identified above which injury would have predicted to occur with reasonable certainty.

Finally, herein we improve the reliability of processes used to identify the injury risk curves and thresholds. In most of the previous TBI metric studies, a single training dataset was used to both develop injury curves and assess the discriminatory power of the metrics without any independent and/or cross-validation of the prediction performance on separate testing dataset(s), due to the limited amount of TBI data available with information on both the kinematic inputs and the resulting brain injuries (Giordano and Kleiven 2014b). This “all in” approach was criticized for overfitting small datasets, lacking sufficient evaluation and validation of performance accuracy with new data (Anderson et al. 2007). Recently, in a few TBI studies, a single (Sullivan et al. 2015) or repeated (Zhao et al. 2017) random training–testing splitting approaches have been used to split the data into randomly independent and non-overlapping development and validation groups for analysis. The prediction accuracy obtained from random training–testing splitting can be dependent on the split, especially when using only a single split on a small dataset. Instead, we adopt the repeated *k*-fold cross-validation technique (Caragea et al. 2007; Yadav and Shukla 2016), which is a common approach in data science field. This method can prevent overfitting and result in more reliable injury metrics. Repeated *k*-fold cross-validation technique is preferred over random as all data are used for both training and testing throughout the k iterations, and each datapoint is used for training k − 1 times and for testing exactly once at each repetition (Caragea et al. 2007; Yadav and Shukla 2016).

In this study, we developed an anisotropic axonal embedded pig head multi-scale FEM using tractography analysis and validated its deformations against ex–vivo hemisection experiment. The FEM was used to simulate a set of well-characterized rapid non-impact head rotation pig TBI experiments in which head kinematics were precisely controlled and measured and the induced axonal injury was quantified through histopathology analysis. The outcomes of the simulations were used to examine different FE-derived tissue metrics for predicting TAI following rapid head rotations and to determine the best predictor(s). Repeated *k*-fold cross-validation approach, along with several risk curve analyses and prediction performance criteria, was used to develop and validate the TAI criteria to prevent bias and improve reliability. Finally, the FE-derived tissue injury thresholds were determined and compared with the results from in–vitro and in–vivo studies. The FE-derived tissue injury curves and thresholds developed in this study may be applied to human FEMs with similar axonal tract implementation to improve TBI prediction in humans.

## Methods

### Finite element model

A previously developed FE head model of a 4-week-old piglet was used as a base for this study (Coats et al. 2012; Sullivan et al. 2015). The brain geometry in the model was determined by analyzing consecutive coronal computed tomography (CT) images of brain and brainstem of a non-injured perfusion-fixed ex–vivo 4-week-old piglet (512 × 512 pixels, 1 mm thick, FOV = 15 cm) in MIMICS 9.0 (Materialise, MI). The skull was created by extending the cortical surface of the brain outward, and falx geometry was added based on ex–vivo measurements. The base FEM consisted of brain, falx, and skull that was previously meshed in MSC Patran (MSC Software, Santa Ana, CA). The model also contains two-dimensional linear elastic spring connectors linking the surface nodes of the brain to the skull to mimic the response of the pia-arachnoid connective tissue, cerebral spinal fluid (CSF), and vasculature located between the brain and skull. The stiffness of these connectors, the brain-skull relative motion, and the boundary condition were previously identified and validated against ex–vivo experiments, and the convergence analysis was performed to ensure performance stability for the model (Coats et al. 2012).

**Fig. 1:**
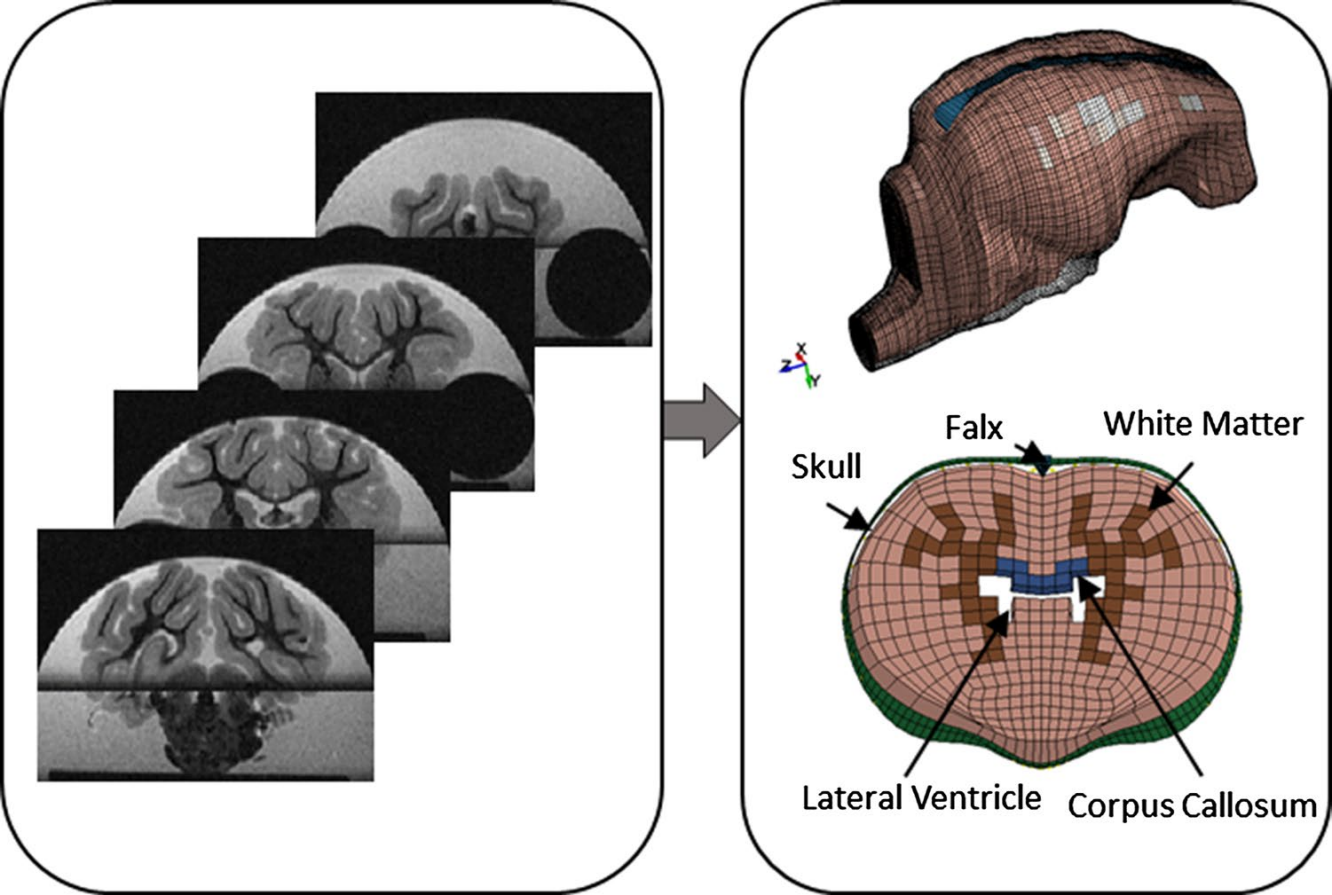
Pig finite element model was enhanced by adding anatomical regions including lateral ventricles, corpus callosum, and white matter tracts

In this study, the base piglet brain FEM was transferred from ABAQUS (Simulia, Providence, RI) to LS-DYNA (v 971 R9.0.1, LSTC, Livermore, CA) and was enhanced by adding anatomical regions including lateral ventricles, corpus callosum, and white matter and embedding axonal structural pathways into the model. Coronal slices of the piglet FEM were registered to the corresponding 4-week-old piglet brain coronal CT images based on appropriate slice increment and the best match of brain shape between FEM and CT slices, and the lateral ventricles, corpus callosum, and white matter regions were segmented (Fig. 1).

**Fig. 2:**
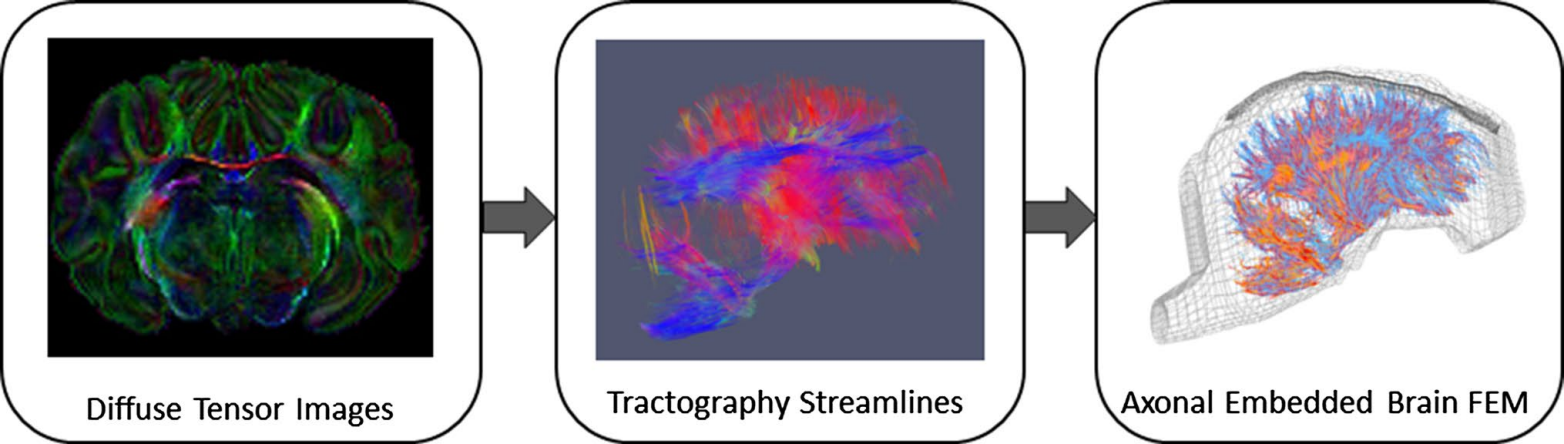
Steps for developing and embedding axonal structural network into pig brain finite element model

The axonal fiber tractography was then performed by processing diffusion tensor imaging (DTI) data of an uninjured perfusion-fixed ex–vivo 4-week-old piglet brain using the Advanced Normalization Tools (ANTs) and Camino software packages to reconstruct fiber streamlines by Euler tracking approach and linear interpolation (Duda et al. 2014). The fractional anisotropy (FA) value of 0.2 and tract turning angle threshold of 60 degree were defined as the streamline stopping criteria in tractography analysis. A step size of 0.2 mm was used for seed map, and a FA value was calculated for each seed point through the streamlines. DTI scan was previously conducted on a 7T Siemens magnet with a 32-channel human head coil (FOV: 64 × 40 × 58 mm^3^, resolution: 0.4 × 0.4 × 1 mm^3^, TR = 400 ms, TE = 60 ms) (Sullivan et al. 2015). The DTI data included a single b = 0 volume and six directional diffusion weighted images acquired with b-value = 1200 s/mm^2^. Tractography streamlines were then used as an input to a custom MATLAB script (V. R2015 MathWorks, Columbia, MD, USA), and a FEM of the axonal bundle structure comprising 5221 tracts and 72,842 1-mm 1-D cable elements was developed. For each axonal fiber element, the average of FA values of streamline seed points along the element was assigned to the element. The axonal fiber elements were then clustered to eight groups depends on the FA value of the element as shown with different colors in Fig. 2. The percentage frequency distribution and range of FA of each group are given in Table 1. The FEM of axonal fiber structure was then incorporated into the piglet brain FEM as embedded elements using the *CONSTRATNED_BEAM_IN_SOLID Keyword in LS-DYNA (v 971 R9.2.0, LSTC, Livermore, CA). This keyword constrains both acceleration and velocity of the axonal fiber structures to the brain solid elements which serve as the master component. A schematic summarizing the workflow of this step is shown in Fig. 2.

**Table 1:** The fractional anisotropy (FA) values and percentage frequency distribution of eight groups of the axonal fiber elements

Group ID	FA values	% of elements in each group
1	0.2–0.3	2.02
2	0.3–0.4	23.78
3	0.4–0.5	30.55
4	0.5–0.6	22.83
5	0.6–0.7	13.76
6	0.7–0.8	5.11
7	0.8–0.9	1.41
8	0.9–1	0.52

### Material property

For material property, considering the microstructural characteristics of white matter, the brain tissue was decomposed into myelinated axons and a mostly isotropic matrix that has similar material property to that of gray matter. The isotropic brain tissue matrix was modeled with solid elements, and the axonal fiber bundles were explicitly modeled using 1-D cable elements. Both brain tissue and axonal fiber were modeled using hyper-viscoelastic material models and implemented in LS-DYNA using a user-defined material model (Wu et al. 2019). For the brain tissue matrix material, the isotropic hyperelastic strain energy density function is (Wu et al. 2019):

1 $$W = \frac{G}{2}\left( {\tilde{I}_{1} - 3} \right) + K\left( {\frac{{J^{2} - 1}}{4} - \frac{1}{2}\ln \left( J \right)} \right) + \frac{{k_{1} }}{{2k_{2} }}\left( {e^{{k_{2} \tilde{E}_{a}^{2} }} - 1} \right)$$

2 $$\tilde{E}_{a} = k\left( {\tilde{I}_{1} - 3} \right)$$

which is an isotropic expression of the Holzapfel–Gasser–Ogden (HGO) model (Gasser et al. 2005) where $\tilde{I}_{1}$ is the first invariant of the isochoric right Cauchy–Green deformation tensor and $J = \det F$ is the volume change ratio. *G* is the shear modulus, *K* is the bulk modulus, *k*_1_ is a stress-like parameter, and *k*_2_ is a dimensionless parameter. The strain energy density function for the axonal fiber is formulated as (Wu et al. 2019):

3 $$W = \frac{{k_{1} }}{{2k_{2} }}\left( {e^{{k_{2} \tilde{E}_{a}^{2} }} - 1} \right)$$

4 $$\tilde{E}_{a} = \kappa \left( {\tilde{I}_{1} - 3} \right) + \left( {1 - 3\kappa } \right)\left( {\tilde{I}_{4a} - 1} \right)$$

5 $$\tilde{I}_{4a} = \tilde{C}:n_{0a} \otimes n_{0a}$$

which is also based on the Holzapfel–Gasser–Ogden (HGO) model (Gasser et al. 2005). $\tilde{C}$ is the deviatoric component of the right Cauchy–Green deformation tensor, and *n*_0*a*_ is the fiber bundle direction unit vector. The dimensionless structure parameter $\kappa$ accounts for the orientation distribution of the axons in a voxel-scale fiber bundle and can be related to FA values of the fiber bundle elements through Eq. (1) (Giordano and Kleiven 2014a; Wright et al. 2013), by assuming similarity between mechanical anisotropy and diffusion anisotropy.

6 $$\kappa = \frac{1}{2}\frac{{ - 6 + 4{\text{FA}}^{2} + 2\sqrt {3{\text{FA}}^{2} - 2{\text{FA}}^{4} } }}{{ - 9 + 6{\text{FA}}^{2} }}$$

For the lower limit of $\kappa$ ($\kappa = 0$, equivalently, FA = 1), axons are perfectly aligned, and for the upper limit of κ ($\kappa = \frac{1}{3}$, equivalently, FA = 0), axons are randomly oriented and isotopically distributed. The range of FA of each group of axonal fiber bundles are given in Table 1. In this model, the axonal fibers do not support compression and they only contribute their mechanical strength during tension; thus, their contribution to the overall mechanical response was neglected when they were under compression.

The temporal response of deviatoric stress was modeled using a quasilinear viscoelastic (QLV) mathematical framework (Fung 2013), as the volumetric behavior was assumed to be independent of time.

7 $$\sigma^{\text{d}} \left( t \right) = \mathop \smallint \limits_{0}^{t} \left[ {G_{\infty } + \mathop \sum \limits_{i = 1}^{n} G_{i} e^{{ - \frac{{t - t^{{\prime }} }}{{\tau_{i} }}}} } \right]\frac{{\partial \sigma_{e}^{\text{d}} }}{{\partial t^{{\prime }} }}{\text{d}}t^{{\prime }}$$

where $\sigma_{e}^{\text{d}}$ is the instantaneous deviatoric elastic response and *G* is the normalized or reduced relaxation function. A Prony series with one-time constant (*n* = 1) was chosen to model the relaxation behavior (Rashid et al. 2014). *G*_∞_ and *G*_*i*_ are the steady-state coefficient and normalized relaxation coefficients of the corresponding time decades, respectively, and *τ*_*i*_ are the decay time constants.

The coefficients of the material model of the matrix brain tissue were calibrated based on a published material testing data of pig brain tissue (Rashid et al. 2014). The calibration process was performed through a generalized reduced gradient nonlinear optimization (Excel Solver^®^, Microsoft^®^, Redmond, WA) to minimize the sum of squared error (SSE) between the experimental data and model predicted stress.

To determine the material properties of axonal fiber bundle, the effective stiffness ratio of *R*_*v*_ between the fiber constitutive model and the brain matrix constitutive model was used with the following formulation:

8 $$R_{v} = \frac{R}{{\gamma_{V} }} , R = \frac{{G_{\text{fiber}} - G_{\text{matrix}} }}{{G_{\text{matrix}} }}$$

where *γ*_*V*_ and $\frac{{G_{\text{fiber}} }}{{G_{\text{matrix}} }}$ are the volume fraction ratio and the stiffness ratio of axonal fiber bundle to the brain tissue matrix, respectively. In the HGO material model used in previous studies, the strain energy density functions for the axonal fiber and isotropic brain matrix are coupled and therefore the same volume sizes were considered for both. In this study, the *R*_*v*_ ratio was used to modify the original HGO material model to accommodate for potential excessive material stiffness and volume redundancy associated with the embedded element modeling approach. The stiffness ratio of 3 ($\frac{{G_{\text{fiber}} }}{{G_{\text{matrix}} }} = 3$) and axonal fiber volume fraction of 0.5 ($\gamma_{V} = 0.5$) experimentally identified by Arbogast and Margulies (1999) for pig brain were used in this study. The detailed material properties of axonal fiber bundles, and brain tissue matrix as well as skull, lateral ventricle, skull-brain connectors, and falx that were employed in the model are given in Table 2.

**Table 2:** Material models and properties used in the anisotropic axonal embedded pig brain finite element model

Anatomical part	Material model	Material properties	References
Brain tissue matrix (White matter, corpus callosum, whole brain)	HGO hyperelastic and quasilinear viscoelastic	$G = 3.0478\,{\text{kPa}}$ $K = 2.19\,{\text{GPa}}$ $k_{1} = 35.767\,{\text{kPa}}$ *k*_2_ → 0 $G_{1} = 0.8909\,{\text{kPa}}$ *G*_∞_ = 0.109 kPa $\tau_{1} = 0.035\,{\text{s}}$ ρ = 1.04 g/cm^3^	Coefficients were calibrated in this study based on the experimental tests performed by Rashid et al. (2014)
Axonal fibers	HGO Hyperelastic and quasilinear viscoelastic	*k*_1_ = 43.432 kPa *k*_2_ → 0 *κ* depending on FA values	Properties were determined in this study based on the volume fraction ratio and the stiffness ratio of axonal fiber bundle to the brain tissue matrix experimentally identified by Arbogast and Margulies (1999)
Falx	Elastic	*E* = 15,000 kPa *υ* = 0.45 *ρ* = 1.13 g/cm3	Sullivan et al. (2015)
Lateral ventricle	Kelvin–Maxwell linear viscoelastic	*G*_0_ = 0.5 kPa *G*_∞_ = 0.1 kPa $\tau_{1} = 0.0125\,{\text{s}}$ $K = 2.19\,{\text{GPa}}$ ρ = 1.04 g/cm^3^	Mao et al. (2013)
Brain-skull connector	Elastic spring	$k = 3460\,{\text{N}}/{\text{mm}}$	Sullivan et al. (2015)

### Validation of pig FEM response against ex–vivo hemisection experiment

To validate the deformation response of the pig FEM and the anisotropic brain material properties employed in the model, the brain strain response obtained from the FEM simulations was compared with the brain deformations measured in ex–vivo hemisection experiment that was previously performed in a high strain and strain rate condition.

**Fig. 3:**
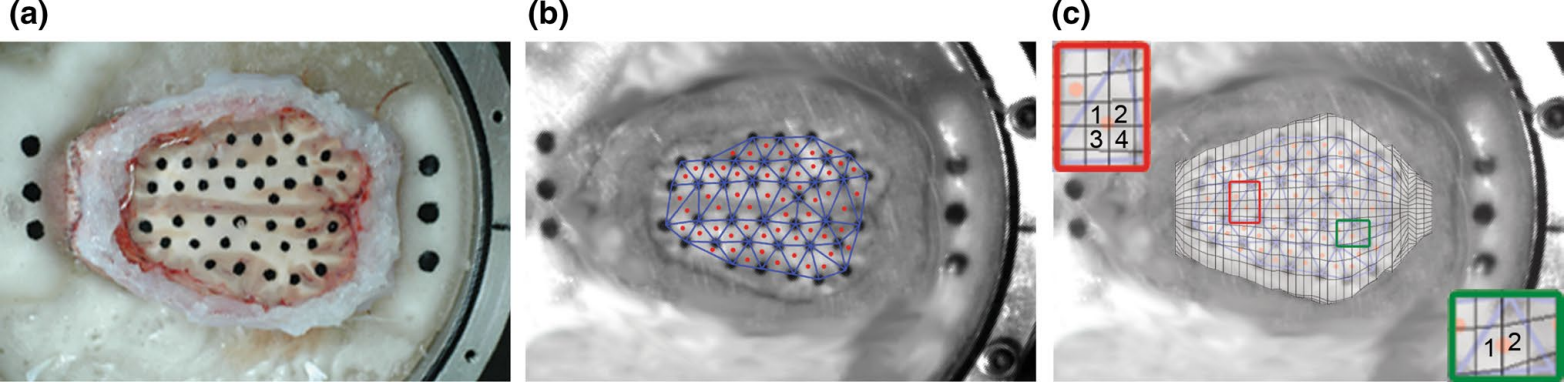
Images of **a** the surface of the head of a 4-week-old pig illustrating ink marks on the brain tissue, **b** a frame from high-speed video demonstrating ink marks isolated by the MATLAB script and triads (blue lines) and their centroids (red dots) that were used for strain calculation, and **c** overlapped FEM top surface and hemisection brain image from high-speed video and examples of how the corresponding elements for triad centroids were selected

In the ex–vivo hemisection experiment as described in detail in Ibrahim et al. (2010a) and Sullivan et al. (2015), a head of 4-week-old piglet obtained immediately after killing was transected in a horizontal plane just above the supraorbital margin and potted into a cylindrical canister. Then, ink markers were placed on the cut brain surface to visualize brain tissue motion (Fig. 3a), a plexiglas cover plate placed on the cut brain surface, and 1-mm space between the brain surface and cover plate was filled with clear lubricant to ensure a frictionless boundary condition. The canister was then mounted onto a HYGE pneumatic actuator system (Bendix Corporation) and was rotated 65**°** at 50 rad/s. The ink markers were tracked with a high-speed digital camera (HG TH, Redlake Tallahassee, FL; resolution of 0.4 mm/pixel) at 2500 fps as shown in Fig. 3b. The velocity trace was measured at 10,000 Hz using two angular velocity transducers (Model ARS-06, ATA Inc., Albuquerque, NM) attached to the actuator side arm. The brain markers were placed into groups of three to form a set of triads (Fig. 3b), and the strain of the center of triads was computed using a custom MATLAB script (Ibrahim et al. 2010a; Sullivan et al. 2015).

**Fig. 4:**
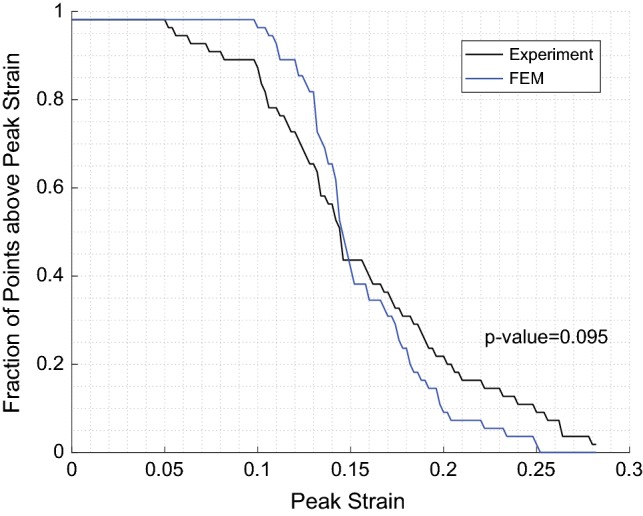
The strain cumulative distribution curves extracted from the hemisection finite element simulation (blue) and ex–vivo hemisection experiment (black)

A hemisection FEM of 4-week-old piglet brain was created by making a horizontal brain transection in the three-dimensional geometries of the pig FEM similar to the one in the ex–vivo hemisection physical model. Then, the measured rotational velocity trace from the hemisection experiment was used as the input loading conditions to reproduce the experiment. The triad centroids on the brain surface of the ex–vivo hemisection physical model were spatially matched to the closest corresponding element centroids in the pig FEM and if a triad centroid did not correspond to the exact location of an element centroid, up to four surrounding elements, which overlapped with the triad and their resultant centroid was close to the triad centroid, were selected as shown in Fig. 3c, and the FEM strain at the triad centroid location was interpolated. Then, the strain cumulative distribution curves extracted from the hemisection FE simulation and experiment were generated (Fig. 4). The Kolmogorov–Smirnov (KS) goodness-of-fit test for continuous distributions was performed between the two cumulative curves to ensure that the two curves were not statistically different (*p* value of > 0.05). Relatively good statistical correlation (*p* value = 0.095) was observed between the pig brain deformation response derived from the newly developed axonal tract embedded FEM and experimentally derived deformation (Fig. 4) with no need for further material property adjustment.

### Animal studies

Once the axonal tract embedded pig brain FEM was developed and validated, the next step was to evaluate the capability of this FEM to predict TAI and develop tissue deformation TAI metrics. To that end, a well-characterized rapid non-impact head rotation (RNR) pig TBI model with biomechanical and neuropathology fidelity to human TBI was used (Margulies et al. 2015). This model produces a purely inertial non-impact head rotation in different anatomical planes at controlled rotational acceleration and velocity levels using the same HYGE pneumatic actuator system as in the ex–vivo hemisection experiment. This pig TBI model creates TAI which can be precisely quantified by immunohistopathology analysis.

For this study, a dataset containing 26 four-week-old and 16 two-month-old pigs that were received a single, rapid non-impact head rotation in the axial or sagittal plane was selected. All protocols for these experiments were approved by the Institutional Animal Care and Use Committee of the University of Pennsylvania, where these experiments were previously conducted. This selected dataset contains a wide range of peak angular velocity (89.54–203.14 rad/s) and peak angular acceleration (18.43–72.36 krad/s^2^). The details about the number of animals at each age group and rotational direction along with the range of peak angular velocity and peak angular acceleration used in these experiments are given in Table 3.

**Table 3:** Summary of the animal dataset used in this study

Pig dataset	Rotational direction	Peak angular velocity (rad/s)	Peak angular acceleration (krad/s^2^)
4 weeks *N* = 26	Axial/Horizontal = 9	113.2–202.9	25.41–61.28
	Injured = 5		
	Non-injured = 4		
	Sagittal = 17	118.3–145.9	30.09–100.92
	Injured = 16		
	Non-injured = 1		
2 months *N* = 16	Axial/Horizontal = 10	106.9–163.9	15.09–57.04
	Injured = 6		
	Non-injured = 4		
	Sagittal = 6	102.7–154.5	18.44–53.55
	Injured = 2		
	Non-injured = 4		

These animals were killed at 6 hours post-injury, and their brains were perfusion-fixed and sectioned in 3-mm coronal slices. Sections were then photographed, cut into 6-µm-thick slices, and stained for beta-amyloid-precursor-protein (βAPP), and areas of TAI identified by βAPP immunostaining analysis were marked. The cumulative sum of marked areas of TAI over the whole brain was used to calculate the axonal injury volume (AIV) which indicates the severity of the axonal injury. The range of AIV calculated for this dataset is 0.02% to 1.65% which represents levels of TBI from no/very minor to mild TBI. After injury and recovering from anesthesia, the animals were returned to their cages without need for further clinical care. Animals with AIV levels < 0.26% were shown to have clinically undetectable injury with no significant behavioral or cognitive deficits (Naim et al. 2010) and thus were considered as non-injured cases for the purpose of TAI metric development in this study. In summary, the animal dataset used in this study included 13 non-injured and 29 injured pigs.

### Simulation of animal experiments

Many studies have found that rapid deformation of brain tissue specially along the axonal fibers can produce axonal injury (Bain and Meaney 2000; Bain et al. 2001; Cullen et al. 2007; Guruprakash 2011; LaPlaca et al. 2005; Morrison et al. 2000; Shi and Whitebone 2006; Singh et al. 2009; Smith and Meaney 2000; Smith et al. 1999). Therefore, the FE model was used to compute the axonal and brain tissue deformation during rapid head rotations in the pig TBI experiments to investigate the relationship between these deformations and resulting TAI. To that end, the measured rotational velocity time-histories of the 42 selected pig experiments were used as input loading conditions for the FE simulations to reproduce the pig experiments. The anisotropic pig brain FEM as explained previously in this section was used as the base for the FE simulations and scaled according to the brain mass of each animal using uniform, isometric mass scaling approach with the scale factor in the following form (Untaroiu et al. 2007):

9 $$\lambda_{x} = \lambda_{y} = \lambda_{z} = \left( {\frac{{m_{\text{scaled}} }}{{m_{\text{base}} }}} \right)^{{\frac{1}{3}}}$$

The uniform mass scaling was used in this study because the details of the brain geometries were not available for the animals in this dataset. However, a separate dataset containing 7 four-week-old and 7 two-month-old pigs, in which the brain geometries were available, was used to determine the geometry variations across subjects and examine how well the uniform scaling works. In this dataset, the anterior–posterior (AP), lateral (L), and superior–inferior (SI) dimensions of the brain were measured. The coefficients of variation (CV = standard deviation/average) for the ratios of these dimensions (AP/L and SI/L) were less than 6% within each age group and both combined, suggesting that uniform mass scaling can be used for scaling in this study. All simulations were performed using LS-DYNA explicit, and model responses were output at every 0.1 ms. Simulations were run longer than angular velocity signals to let the brain to return to its original state. From each simulation, the first principal strain of every brain element and the axial logarithmic strain of every axonal embedded element were extracted at each output state, and their maximum values during the entire simulation were calculated as the maximum principal strain (MPS) and the maximum axonal strain (MAS), respectively. The element-wise strain rate was calculated as the first-order discrete derivative of strain between time points, and the element-wise product of strain and strain rate (SxSR) was calculated as element-wise multiplication of strain by its strain rate for each time point. For each simulation, the maximum value of the first principal strain rate (MPSR) and the maximum value of the product of the first principal strain and its strain rate (MPSxSR) for every brain element as well as maximum value of axonal strain rate (MASR), and maximum value of the product of axonal strain and its strain rate (MASxSR) for every axonal fiber embedded element were also calculated over the entire duration of simulation. The 95th percentile values of MPS, MPSR, and MPSxSR of the brain elements, and the 95th percentile values of MAS, MASR, and MASxSR of the axonal fiber elements were extracted from all 42 simulations and used as potential TAI predictors (Table 4). The maximum 95th percentile was selected instead of the 100th percentile value to eliminate any possible computational artifacts from the analysis (Panzer et al. 2012). All the analyses were also performed using 100th percentile values and showed similar or slightly worse prediction performance than 95th percentile results. These six metrics were examined as TAI predictors because several experimental studies of TAI performed in the isolated nerve fibers (Bain and Meaney 2000; Bain et al. 2001) or axons (Smith et al. 1999), brain slices (Morrison et al. 2000), and axonal or neuronal cultures (Cullen et al. 2007; LaPlaca et al. 2005) showed that the degree of axonal morphology and electrophysiological impairment of TAI were directly related to the magnitude and rate of brain and/or axonal stretch.

**Table 4:** Summary of the 12 selected FE-derived deformation-related metrics used in this study to predict the presence or absence of traumatic axonal injury

Predictor candidate	Description
MPS	95th percentile maximum principal strain of the brain tissue elements
MPSR	95th percentile maximum principal strain rate of the brain tissue elements, the strain rate for each element was calculated at every 0.1 ms time step as the discrete derivative of the 5-point moving-window-average smoothed first principal strain signal for each element
MPSxSR	95th percentile maximum principal strain times strain rate of brain tissue elements, strain times strain rate value for each element was calculated at every 0.1 ms time step by multiplying the first principal strain and its strain-rate value
MAS	95th percentile maximum axial logarithmic strain of the axonal fiber embedded elements
MASR	95th percentile maximum logarithmic strain rate of the axonal fiber embedded elements calculated similar to MPSR
MASxSR	95th percentile maximum logarithmic strain times strain rate of the axonal fiber embedded elements calculated similar to MPSxSR
BF-MPS_30_	Volume fraction of brain elements that passed MPS of 30, similar to CSDM30
BF-MPSR_120_	Volume fraction of brain elements that passed MPSR of 120 s^−1^
BF-MPSxSR_28_	Volume fraction of brain elements that passed MPSxSR of 28 s^−1^
AF-MAS_13_	Fraction of axonal embedded elements that passed MAS of 13
AF-MASR_70_	Fraction of axonal embedded elements that passed MASR of 70 s^−1^
AF-MASxSR_7.5_	Fraction of axonal embedded elements that passed MASxSR of 7.5 s^−1^

**Fig. 5:**
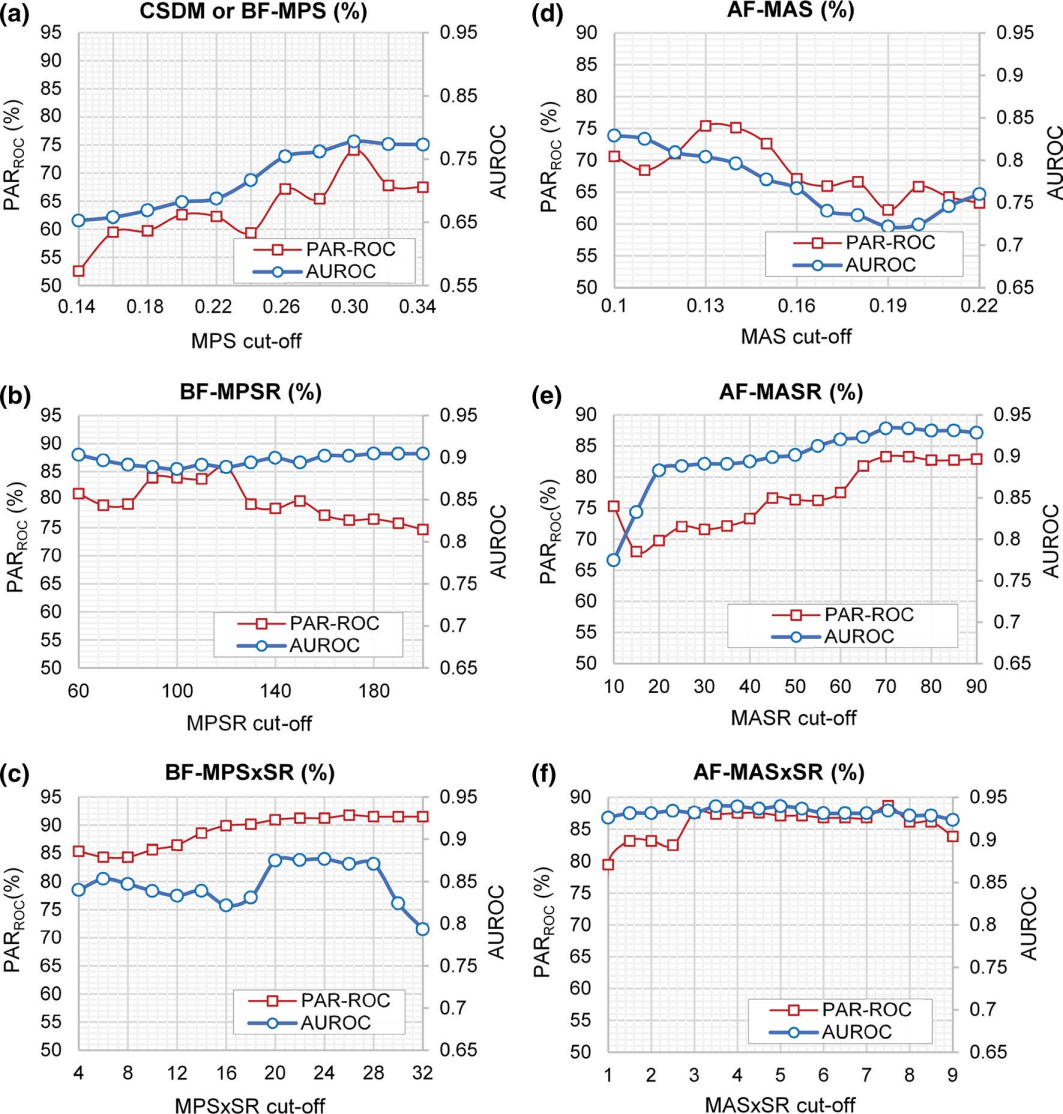
The overall prediction accuracy rate (PAR_ROC_) and AUROC based on fraction-based metrics including **a** BF-MPS, **b** BF-MPSR, **c** BF-MPSxSR, **d** AF-MAS, **e** AF-MASR, and **f** AF-MASxSR using different selected MPS, MPSR, MPSxSR, MAS, MASR, MASxSR cutoff values to determine the optimal cutoff values resulting in the highest PAR_ROC_ and AUROC for each of the fraction-based metrics

While magnitude-based injury metrics such as MAS, MASR, MASxSR, MPS, MPSR, and MPSxSR have been shown to correlate with the degree of axonal injury as stated above, the extent of injury is mostly evaluated by the percentage of axons and/or neuronal cells that are damaged (Bar-Kochba et al. 2016; Cullen et al. 2007). For this reason, we examined 6 new metrics that are based on the fraction of the axonal fibers exceeding a selected MAS, MASR, or MASxSR cutoff value, AF-MAS, AF-MASR, and AF-MASxSR, respectively, or the volume fraction of the brain exceeding a selected MPS, MPSR, or MPSxSR cutoff value, BF-MPS, BF-MPSR, and BF-MPSxSR, respectively. For example, AF-MAS_13_, AF-MASR_70_, and AF-MASRxSR_7.5_ represent the fraction of the axonal fiber elements that passed MAS of 0.13, MASR of 70 s^−1^, and MASRxSR of 7.5 s^−1^, respectively. Similarly, BF-MPS_30_, BF-MPSR_120_, and BF-MPSRxSR_28_ represent the volume fraction of the brain elements that passed MPS of 0.30, MPSR of 120 s^−1^, and MPSRxSR of 28 s^−1^, respectively. For each of the six fraction-based metrics, wide ranges of possible cutoff threshold values of MPS, MPSR, MPSxSR, MAS, MASR, and MASxSR varied between 0.14 and 0.34, 60 and 200 (/s), 4 and 32 (/s), 0.1 and 0.22, 10 and 90 (/s), and 1 and 9 (/s), respectively, were examined, and the corresponding BF-MPS, BF-MPSR, BF-MPSxSR, AF-MAS, AF-MASR, and AF-MASxSR values for all 42 dataset were calculated. Then, the area under the ROC curve (AUROC) and the overall prediction accuracy rate (PARROC), that will be described in more detail in the next section, were calculated for each of the six fraction-based metrics at every selected threshold level (Fig. 5). AUROC and PAR_ROC_ were used to evaluate the TAI prediction performance of these metrics. For each of these six fraction-based metrics, among all the threshold values examined (Fig. 5), the one that led to the highest PAR_ROC_ and AUROC was selected as the optimal cutoff threshold value. Between these two TAI prediction performance criteria, when their results are not in agreement, priority was given to PAR_ROC_ for determining the optimal cutoff threshold. A similar metric in the literature is the cumulative strain damage measure (CSDM) that is the fractional volume of the brain that exceeds a specified maximum principal strain threshold (e.g., BF-MPS_30_ is equivalent to CSDM30). CSDM was previously introduced as a potential predictor of TBI (Bandak and Eppinger 1994) and demonstrated that can be linked to the severity of TBI (Kimpara and Iwamoto 2012). Similarly, the fraction-based metrics introduced in this study can provide an estimation of the volume of brain injury. The fraction of the axonal bundles/brain predicted as damaged through FE simulations are required to be above a threshold value for the subject to be classified as injured using these 6 fraction-based metrics. The 12 selected FE-derived metrics used for TAI prediction in this study are summarized in Table 4.

For each animal, the volume fraction of the brain that predicted as injured (damaged volume fraction, DVF) using each of the 6 fraction-based metrics was also calculated. The DVFs predicted by these 6 FE-derived metrics were compared to the AIV identified through histopathology analysis for injured (*n* = 29) and non-injured animals (*n* = 13). For the brain-tissue-related metrics, the DVFs are equivalent to the values of BF-MPS, BF-MPSR, and BF-MPSxSR. For the axonal-related metrics, considering the axonal fiber-brain matrix fraction of ~ 0.5 used in our FEM [referring to the experimental study by Arbogast and Margulies (1999)], the fraction of axonal fiber predicted as injured (AF-MAS, AF-MASR, and AF-MASxSR) multiplied by 0.5 gives an estimation of the DVFs. To get the axonal fiber-brain matrix fraction of 0.5, the radius of 0.37 mm was used for the cross section of the axonal fiber elements in this study. Therefore, the DVFs based on the axonal-related metrics represent the volume within a radius of 0.37 mm around the axonal tracts that their MAS, MASR, or MASxSR values passed the selected injury thresholds.

### Development of TAI metrics

For each of the 12 selected TAI predictor candidates, 42 data points were computed from the FE simulations and a binary classifier was assigned to each data point to designate whether the animal was injured (1) or non-injured (0). Binary logistic regression (BLR) analysis, survival analysis with Weibull distribution, and receiver operating characteristic (ROC) curve analysis were then performed to examine and compare the abilities of the selected parameters to predict the presence or absence of clinically detectable TAI. These are the most common statistical methods currently used to develop injury risk curves and determine injury threshold values in the biomechanical field. The survival analysis with Weibull distribution is recommended over BLR analysis, especially when the distribution of the data is not normal which can lead to nonzero risk at zero value of predictor (Petitjean and Trosseille 2011). BLR risk curves (Eq. 10) and Weibull risk curves (Eq. 11) have distribution functions of the following form:

10 $$P\left( x \right) = \frac{{e^{a + bx} }}{{1 + e^{a + bx} }}$$

11 $$P\left( x \right) = \left\{ {\begin{array}{*{20}c} {1 - e^{{ - (\frac{x}{\alpha })^{\beta } }} } & {x \ge 0} \\ 0 & {x < 0} \\ \end{array} } \right\}$$

where *P*(*x*) is the probability of TAI for the given value x of the predictor candidate, variables *a* and *b* are the regression coefficients for BLR risk curves, and α and β are scale and shape parameters for Weibull risk curves. The quality of fit for the BLR risk curves evaluated using the adjusted *R*-squared value (Mittlböck and Schemper 1998), which represent the quality of fit of the risk curves with a larger value indicating better fit and higher correlation with TAI. The Weibull risk curves were compared using the Akaike Information Criterion (AIC_Weibull_). Lower AIC_Weibull_ represents better fit model (Petitjean and Trosseille 2011).

ROC curves were also evaluated by the area under the ROC curves (AUROC), which is a measure of prediction performance with higher AUROC indicating better TAI predictive capability. The threshold values associated with 50% likelihood of injury were extracted from the BLR curves and Weibull risk curves, and the optimal ROC threshold values, optimizing both specificity and sensitivity giving equal weight to both (point on ROC curve closest to the (0, 1)), were extracted from ROC curves. The 50% likelihood and the optimal ROC threshold values were then used to evaluate the prediction performance of the injury metrics by calculating sensitivity, specificity, and overall accuracy rate with the following formulations:

12 $${\text{S}} = {\text{Sensitivity}} = \frac{\text{TP}}{\mathrm{P}}$$

13 $${\text{SP}} = {\text{Specificity}} = \frac{\text{TN}}{\mathrm{N}}$$

14 $${\text{PAR}} = {\text{overall}}\,{\text{prediction}}\,{\text{accuracy}}\,{\text{rate}} = \frac{{{\text{TN}} + {\text{TP}}}}{{{\mathrm{N}} + {\text{P}}}}$$

where true positives (TP) and true negatives (TN) are the number of cases correctly identified as injured and non-injured, respectively. P is the number of real positive (injured) cases (P), and N is the number of real negative (non-injured) cases.

Overall, 12 criteria including AUROC, BLR *R*-squared, AIC_Weibull_, overall prediction accuracy rate (PAR), sensitivity (S), and specificity (SP) based on the optimal ROC thresholds (PAR_ROC_, S_ROC_, and SP_ROC_) and the 50% BLR risk thresholds (PAR_50%-BLR_, S_50%-BLR_, SP_50%-_BLR) and 50% Weibull risk threshold (PAR_50%-Weibull_, S_50%-Weibull_, and SP_50%-Weibull_) were used to evaluate and compare the prediction performances of the 12 metrics. PAR_ROC_ and AUROC were given the highest priority for determining the optimal TAI predictor.

To obtain a more reliable evaluation of prediction performance of the predictor candidates and prevent bias and overfitting of the risk curves, repeated *k*-fold cross-validation (CV) technique was used (Caragea et al. 2007; Yadav and Shukla 2016) in this study. In *k*-fold CV technique, instead of using all data as both the training dataset and the test dataset, the data were partitioned into k equal or nearly equal subsamples. The data for each of the 12 metrics were stratified before partitioning in a way that each subsample had a good representative of the whole dataset in terms of the relative number of injured versus non-injured data points. At each iteration of *k*-fold CV, k-1 subsamples were combined and used as a training dataset to perform BLR, Weibull, and ROC analyses and determine the injury thresholds, and one subsample was left out as testing dataset to evaluate the injury prediction performance by calculating the overall accuracy rate, sensitivity, and specificity. For each partitioning, k iterations were performed by changing the testing subsample one-by-one until every k subsample was used as the testing dataset once. The cross-validation process with *k* = 5 was used in this study that partitioned data to 79–83% as training and 17–21% as testing data at each iteration. A value of *k* = 5 was selected to ensure that the ‘one-in-ten’ rule (requiring 10 data points in each class for one predictive variable) was followed. To improve the reliability of the prediction performance evaluation process even further, 5-fold CV was repeated 50 times, and the data were reshuffled, re-stratified, and re-partitioned before each repetition. For all predictor candidates, the optimal ROC threshold, 50% likelihood thresholds based on BLR and Weibull risk curves, AUROC, AIC_Weibull_, BLR *R*-squared, PAR_ROC_, S_ROC_, SP_ROC_, PAR_50%-BLR_, S_50%-BLR_, SP_50%-BLR_, PAR_50%-Weibull_, S_50%-Weibull_, and SP_50%-Weibull_ results that were obtained at all 250 iterations (50 repetitions of 5-fold CV) were averaged and are reported in Tables 5, 6, and 7. Statistical analyses were performed in MATLAB and R.

**Table 5:** Averages and standard deviations of the results from the binary logistic regression analyses coupled with 50 repeated 5-fold cross-validation analyses (250 iterations) for predicting traumatic axonal injury based on all 12-predictor metrics

	Overall prediction accuracy rate (PAR_50%-BLR_)	Sensitivity (S_50%-BLR_)	Specificity (SP_50%-BLR_)	*R*-squared	50% Likelihood threshold (th_50%-BLR_)
MAS	76% ± 11%	90% ± 10%	44% ± 30%	0.35 ± 0.07	0.12 ± 0.002
MASR (s^−1^)	84% ± 11%	89% ± 15%	74% ± 27%	0.62 ± 0.07	66.45 ± 1.43
**MAS × SR (s**^**−1**^**)**	**87% ± 10%**	**91% ± 10%**	**76% ± 26%**	**0.67 ± 0.07**	**4.87 ± 0.15**
MPS	75% ± 8%	90% ± 8%	42% ± 21%	0.37 ± 0.10	0.29 ± 0.00
MPSR (s^−1^)	82% ± 9%	89% ± 12%	67% ± 22%	0.61 ± 0.11	140.86 ± 3.71
MPSxSR (s^−1^)	86% ± 8%	91% ± 9%	75% ± 19%	0.65 ± 0.09	24.91 ± 1.18
AF-MAS_13_ (%)	75% ± 14%	90% ± 12%	43% ± 30%	0.34 ± 0.07	3.65% ± 0.29%
AF-MASR_70_ (%)	82% ± 10%	82% ± 17%	81% ± 25%	0.59 ± 0.08	4.73% ± 0.52%
**AF-MAS × SR**_**7.5**_**(%)**	**87% ± 10%**	**91% ± 11%**	**77% ± 27%**	**0.67 ± 0.08**	**1.72% ± 0.15%**
BF-MPS_30_ (%)	73% ± 12%	90% ± 12%	34% ± 29%	0.27 ± 0.06	2.49% ± 0.64%
BF-MPSR_120_ (%)	82% ± 13%	89% ± 17%	76% ± 25%	0.52 ± 0.10	8.92% ± 0.68%
BF-MPSxSR_28_ (%)	86% ± 11%	90% ± 11%	77% ± 25%	0.61 ± 0.08	3.01% ± 0.28%

The most TAI predictive metrics are highlighted in bold

**Table 6:** Averages and standard deviations of the results from the ROC curve analyses coupled with 50 repeated 5-fold cross-validation analyses (250 iterations) for predicting traumatic axonal injury based on all 12-predictor metrics

	Overall prediction accuracy rate (PAR_ROC_)	Sensitivity (S_ROC_)	Specificity (SP_ROC_)	AUROC	Optimal ROC threshold (th_ROC_)
MAS	75% ± 14%	75% ± 17%	74% ± 27%	0.81 ± 0.04	0.13 ± 0.005
MASR (s^−1^)	81% ± 12%	84% ± 15%	77% ± 26%	0.93 ± 0.02	68.11 ± 1.24
**MASxSR (s**^**−1**^**)**	**88% ± 11%**	**86% ± 14%**	**92% ± 16%**	**0.93 ± 0.02**	**5.86 ± 0.02**
MPS	75% ± 15%	76% ± 17%	69% ± 18%	0.81 ± 0.05	0.30 ± 0.00
MPSR (s^−1^)	85% ± 11%	85% ± 14%	84% ± 16%	0.91 ± 0.04	146.47 ± 7.10
MPSxSR (s^−1^)	81% ± 12%	81% ± 16%	82% ± 19%	0.91 ± 0.03	29.31 ± 1.98
AF-MAS_13_ (%)	75% ± 14%	76% ± 19%	74% ± 28%	0.81 ± 0.04	5.01% ± 0.12%
AF-MASR_70_ (%)	84% ± 15%	86% ± 15%	77% ± 27%	0.93 ± 0.02	4.34% ± 0.38%
**AF-MASxSR**_**7.5**_**(%)**	**90% ± 10%**	**89% ± 13%**	**91% ± 20%**	**0.93 ± 0.02**	**2.58% ± 0.17%**
BF-MPS_30_ (%)	74% ± 16%	79% ± 23%	62% ± 28%	0.78 ± 0.04	4.18% ± 0.57%
BF-MPSR_120_ (%)	86% ± 13%	90% ± 18%	77% ± 23%	0.89 ± 0.04	9.12% ± 2.54%
BF-MPSxSR_28_ (%)	87% ± 11%	86% ± 13%	90% ± 19%	0.92 ± 0.03	4.43% ± 0.38%

The most TAI predictive metrics are highlighted in bold

**Table 7:** Averages and standard deviations of the results from the survival analysis with Weibull distribution coupled with 50 repeated 5-fold cross-validation analyses (250 iterations) for predicting diffuse axonal injury based on all 12-predictor metrics

	Overall prediction accuracy rate (PAR_50%-Weibull_)	Sensitivity (S_50%-Weibull_)	Specificity (SP_50%-Weibull_)	AIC	50% Likelihood threshold (th_50%-Weibull_)
MAS	76% ± 12%	89% ± 13%	45% ± 29%	36 ± 3	0.12 ± 0.002
MASR (s^−1^)	83% ± 12%	86% ± 15%	75% ± 27%	26 ± 3	67.1 ± 1.5
**MASxSR (s**^**−1**^**)**	**86% ± 10%**	**90% ± 11%**	**76% ± 25%**	**23 ± 4**	**4.9 ± 0.1**
MPS	75% ± 13%	89% ± 11%	41% ± 29%	36 ± 3	0.29 ± 0.00
MPSR (s^−1^)	81% ± 13%	87% ± 16%	70% ± 30%	27 ± 3	142.1 ± 2.9
MPSxSR (s^−1^)	85% ± 11%	90% ± 11%	75% ± 27%	24 ± 3	25.1 ± 0.8
AF-MAS_13_ (%)	75% ± 11%	89% ± 12%	42% ± 29%	36 ± 3	3.5% ± 0.3%
AF-MASR_70_ (%)	83% ± 12%	89% ± 15%	70% ± 29%	25 ± 3	5.3% ± 0.6%
**AF-MASxSR**_**7.5**_**(%)**	**86% ± 11%**	**93% ± 12%**	**70% ± 27%**	**23 ± 3**	**1.5% ± 0.1%**
BF-MPS_30_ (%)	73% ± 11%	89% ± 11%	35% ± 29%	38 ± 2	2.7% ± 0.4%
BF-MPSR_120_ (%)	83% ± 13%	90% ± 9%	68% ± 29%	28 ± 1	7.9% ± 3.9%
BF-MPSxSR_28_ (%)	85% ± 11%	90% ± 12%	74% ± 27%	25 ± 2	2.3% ± 0.3%

The most TAI predictive metrics are highlighted in bold

## Results

Simulations were run for 42 pig TBI experiments using the newly enhanced axonal tract embedded pig brain FEM to determine the optimal FE-derived metrics capable of predicting clinical TAI. Figure 5 shows a summary of the prediction performance for each of the 6 fraction-based metrics for different cutoff values. The prediction performance is evaluated by the average PAR_ROC_ and AUROC from 50-repeated 5-fold CV. For each fraction-based metric, the optimal cutoff values that led to the highest PAR_ROC_ and AUROC were selected. The optimal cutoff values determined were 0.13, 70 (s^−1^), 7.5 (s^−1^), 0.3, 120 (s^−1^), and 28 (s^−1^) for MAS, MASR, MASxSR, MPS, MPSR, and MPSxSR, respectively. Interestingly, these optimal cutoff values were the same or very similar to the thresholds that were obtained for magnitude-based metrics (Tables 5, 6, 7). For BF-MPSxSR, the cutoff values of 20 (s^−1^) to 28 (s^−1^) showed the same prediction capabilities. The cutoff value of 28 (s^−1^) was selected because the actual AIVs obtained from histopathology for these TBI experiments were small (< 2%) and the predicted damaged brain volume fraction for the MPSxSR cutoff value of 28 (s^−1^) (BF-MPSxSR_28_), as expected, was the smallest among the other cutoffs.

**Fig. 6:**
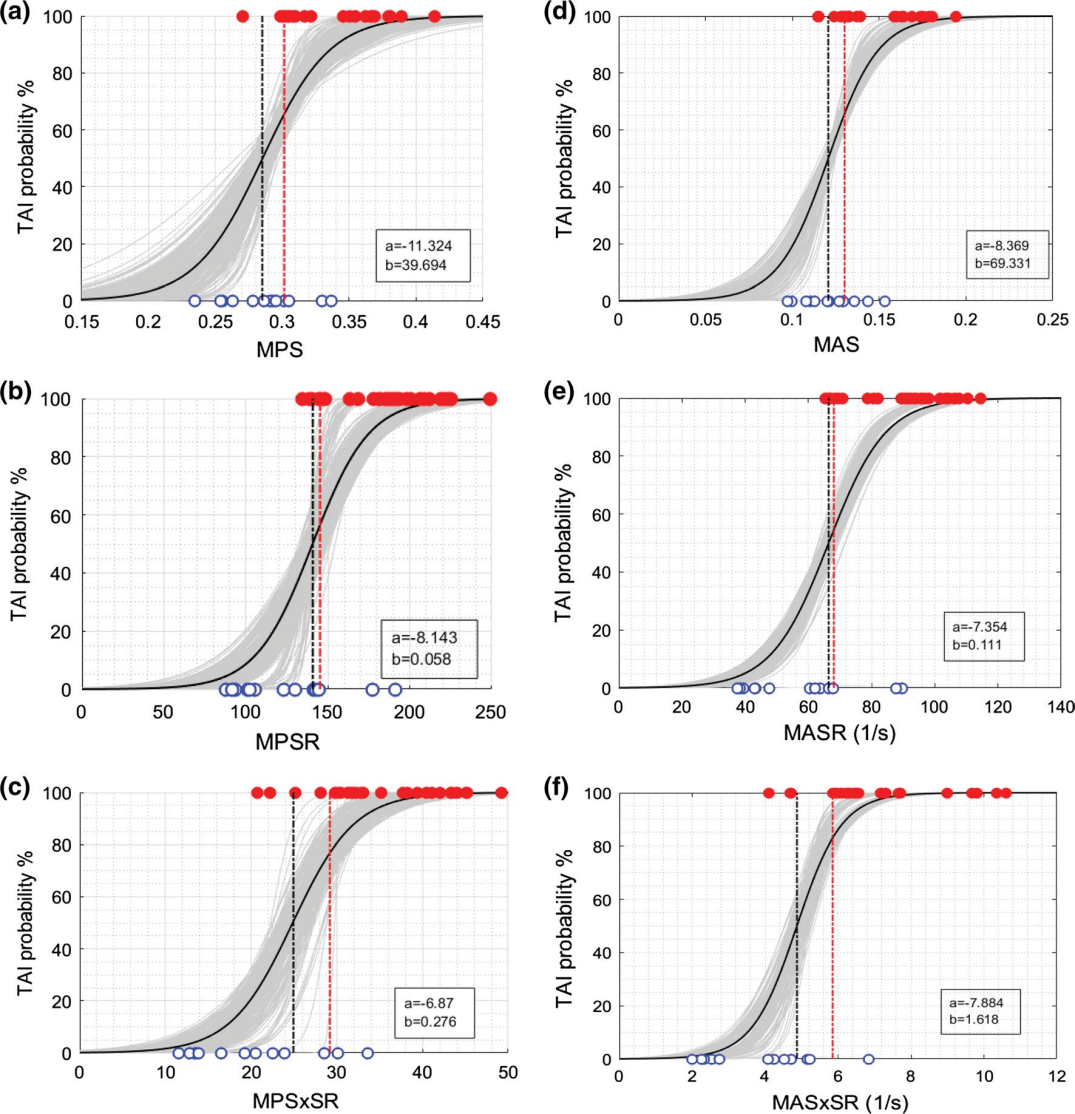
Binary logistic regression (BLR) TAI risk curves based on **a** MPS, **b** MPSR, **c** MPSxSR, **d** MAS, **e** MASR, **f** MASxSR. BLR curves from the 50 repeated 5-fold CV analyses (250 iterations) are shown in gray curves. BLR curve using the whole datapoints (29 injured and 13 non-injured) as the training set is shown in black curve along with associated regression coefficients (a and b in legend) in each graph. The vertical black and red dash-dot lines indicate the 50% BLR risk thresholds and the optimal ROC thresholds, respectively. The depicted thresholds are the average of 50 repeated 5-fold CV analyses

**Fig. 7:**
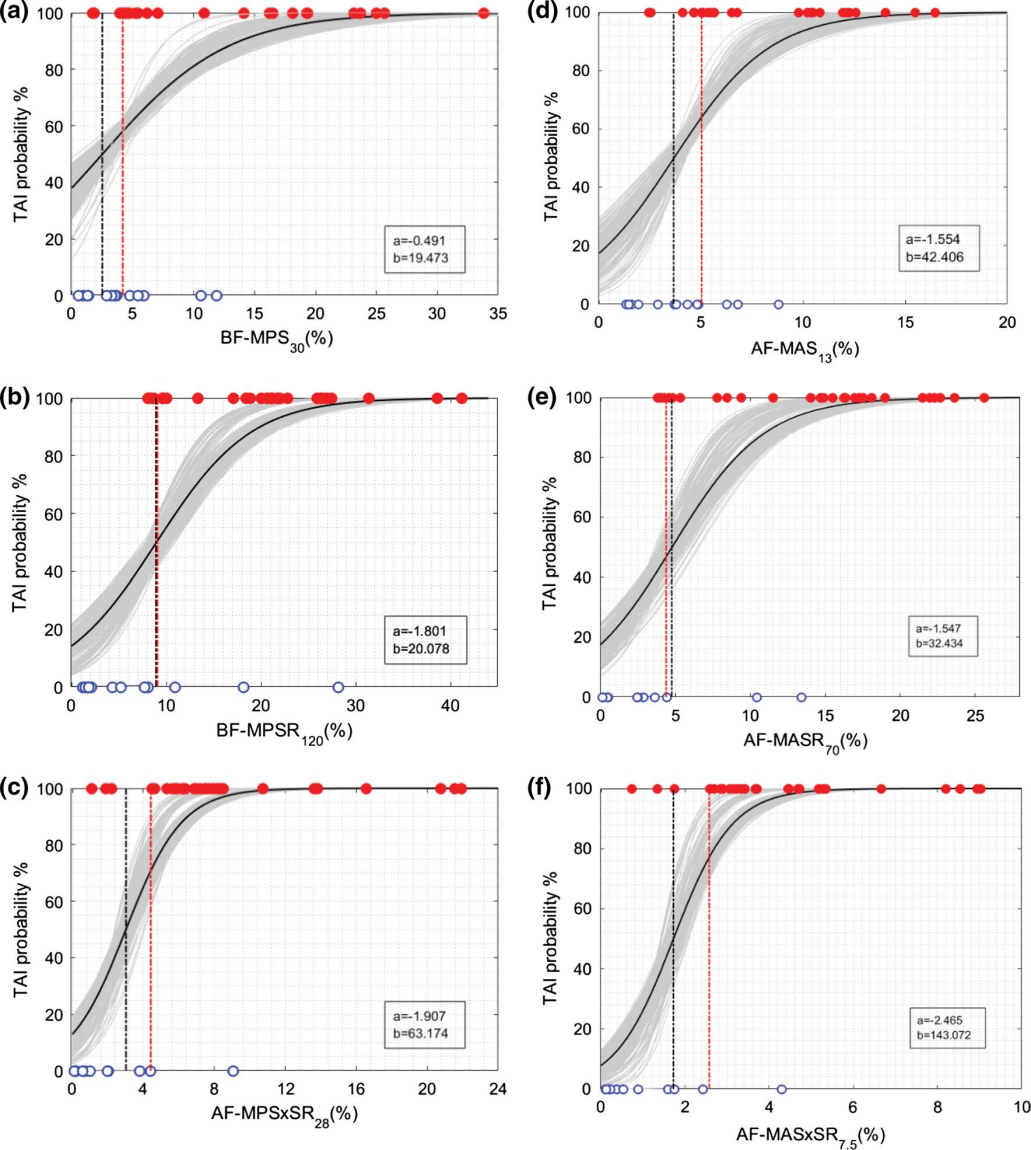
BLR TAI risk curves based on **a** BF-MPS_30_, **b** BF-MPSR_120_, **c** BF-MPSxSR_28_, **d** AF-MAS_13_, **e** AF-MASR_70_, **f** AF-MASxSR_7.5_, BLR curves from the 50 repeated 5-fold CV analyses are shown in gray curves. BLR curve using the whole datapoints (29 injured indicated with red filled markers and 13 non-injured indicated with open blue markers) as the training set is shown in black curve along with associated regression coefficients (a and b in legend) in each graph. The vertical black and red dash-dot lines

**Fig. 8:**
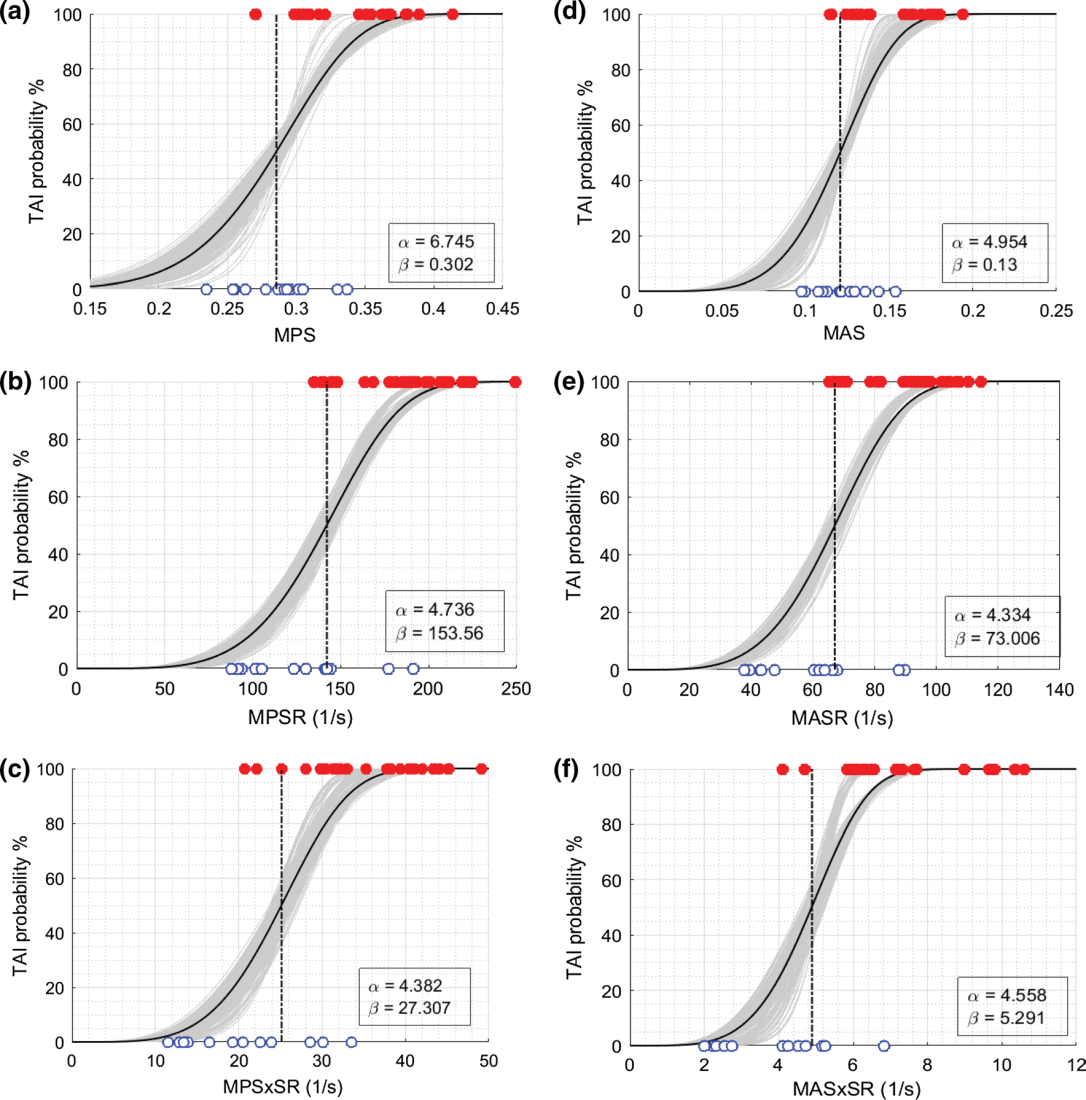
Survival TAI risk curves with Weibull distribution based on **a** MPS, **b** MPSR, **c** MPSxSR, **d** MAS, **e** MASR, **f** MASxSR. Survival risk curves from the 50 repeated 5-fold CV analyses (250 iterations) are shown in gray curves. Survival risk curve using the whole datapoints (29 injured and 13 non-injured) as the training set is shown in black curve along with associated scale and shape coefficients (*α* and *β* in legend) in each graph. The vertical black dash-dot lines indicate the 50% risk level thresholds. The depicted thresholds are the average of 50 repeated 5-fold CV analyses

**Fig. 9:**
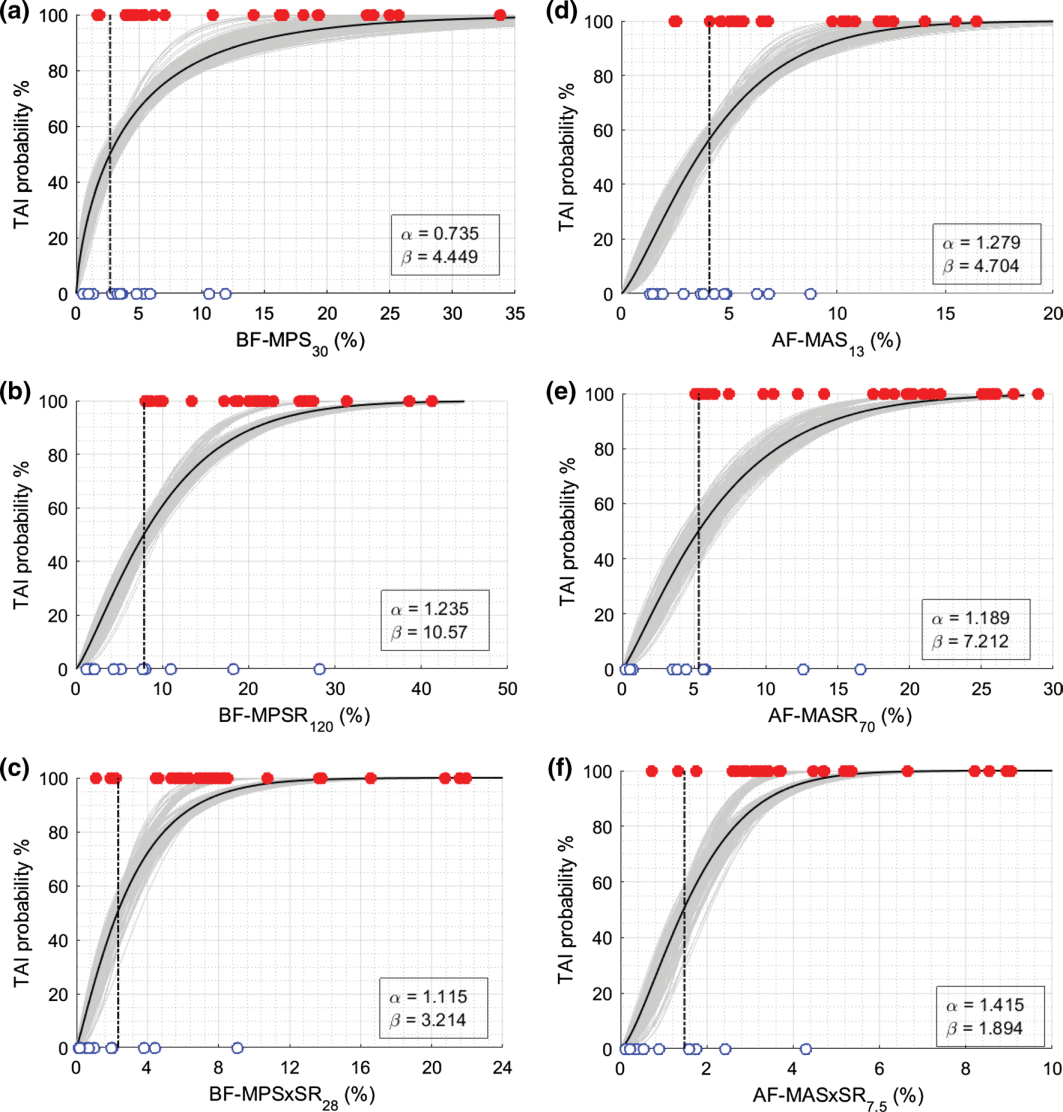
Survival TAI risk curves with Weibull distribution based on **a** BF-MPS_30_, **b** BF-MPSR_120_, **c** BF-MPSxSR_28_, **d** AF-MAS_13_, **e** AF-MASR_70_, **f** AF-MASxSR_7.5_. Survival risk curves from the 50 repeated 5-fold CV analyses (250 iterations) are shown in gray curves. Survival risk curve using the whole datapoints (29 injured and 13 non-injured) as the training set is shown in black curve along with associated scale and shape coefficients (*α* and *β* in legend) in each graph. The vertical black dash-dot lines indicate the 50% risk level thresholds. The depicted thresholds are the average of 50 repeated 5-fold CV analyses

BLR, Weibull, and ROC curve analyses were performed for all 12 metrics, and the average and standard deviation of the results for 50-repeated 5-fold CV are summarized in Tables 5, 6, and 7. The probability curves for TAI prediction developed by BLR analysis and survival analysis with Weibull distribution based on these predictor candidates are also illustrated in Figs. 6, 7, 8, and 9. In each graph, BLR and Weibull probability curves from the 50 repetitions of 5-fold CV (250 gray curves), developed using 79-83% of data in 250 iterations, represent a range of possible probability curve for TAI prediction. For the faction-based metrics, the BLR risk curves (Fig. 7) resulted in nonzero risks at zero value of the predictors (especially for BF-MPS_30_) and therefore the Weibull risk curves (Fig. 9) would be recommended for these metrics. The 12-selected metrics were compared using the 6 prediction performance criteria including PAR_ROC_, PAR_50%-BLR_, PAR_50%-Weibull_, AUROC, AIC_Weibull_, and BLR *R*-squared (Tables 5, 6, 7).

AUROC, which is a measure of discrimination performance on the training datasets, showed average values ≥ 0.78 for all the 12 selected parameters and the average values ≥ 0.89 for the 8 metrics related to strain rate and the product of strain and strain rate. These high AUROC values indicate that any of the 12 parameters can discriminate the subjects that sustained TAI from the non-injured subjects. Also, higher AUROC for the strain rate-related metrics (SR ad SxSR) indicates that these metrics have higher discriminatory power for TAI than strain-based metrics. BLR *R*-squared values, which is a measure of the goodness of fit for the BLR curves on the training datasets, showed relatively high value for MASR, MASxSR, MPSR, MPSxSR and their optimal fraction-based metrics including AF-MASR_70_, AF-MASxSR_7.5_, BF-MPSR_120_, and BF-MPSxSR_28_ (*R*-squared = 0.60-0.67), indicating that there were high correlations between risk of TAI and these eight parameters and low overlap between their values for injured and non-injured cases. Lower values of *R*-squared from BLR analysis for MPS, MAS, BF-MPS_30_, and AF-MAS_13_ were observed (*R*-squared = 0.27-0.37) that indicates poor fit for the BLR curves for these metrics. Also, the lowest AIC_Weibul_, which represent the best risk curve fit, were obtained for MASxSR and AF-MASxSR_7.5_ metrics.

The average overall prediction accuracy rates (PAR_ROC_, PAR_50%-Weibull_, and PAR_50%-BLR_) and sensitivities (S_ROC_, S_50%-Weibull_, and S_50%-BLR_), which represent the prediction performance of the metrics in facing new data, were in the range of 73% to 90% for all the 12 metrics and were particularly high (>=85%) for the 4 metrics related to the product of strain and strain rate, indicating that these metrics are more predictive of TAI than the remaining metrics.

**Fig. 10:**
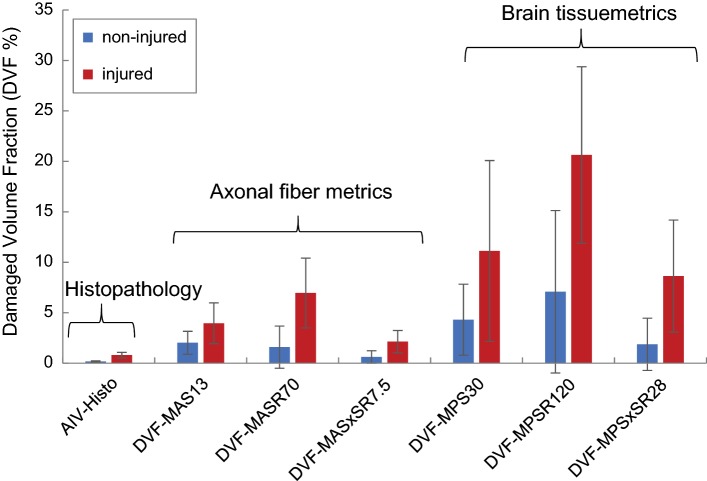
The average volume fraction of the whole brain determined to have axonal damaged through histopathology analyses (AIV-Histo) and predicted as damaged through FE simulations based on axonal fiber elements passed MAS of 13% (DVF-MAS_13_), MASR of 65 s^−1^ (DVF-MASR_70_), or MASxSR of 7.5 s^−1^ (DVF-MASxSR_7_._5_), or based on brain elements passed MPS of 30% (DVF-MPS_30_), MPSR of 120 s^−1^ (DVF-MPSR_120_), or MPSxSR of 28 s^−1^ (DVF-MPSxSR_28_) for 13 non-injured and 29 injured pigs

For each of the 12 selected metrics, the optimal ROC TAI threshold (Table 5, last column) determined from ROC analysis and the 50%` likelihood TAI thresholds (Tables 5 and 7, last column) derived from BLR or Weibull analyses were similar. These thresholds are common and widely used in injury prediction studies and thus were reported in this study. For the magnitude-based metrics, TAI thresholds of the axonal metrics were significantly lower than their corresponding thresholds of the brain tissue metrics, implying that the maximum principal strain direction of brain tissue following rapid rotation is not often aligned with the axonal bundles. The threshold values for the axonal-related fraction-based metrics indicate the percentage of the axonal fibers that are required to be above the cutoff values for the subject to be classified as injured. For instance, the 50% likelihood threshold of AF-MASxSR_7.5_ = 2.3% means that there is 50% probability of sustaining clinical TAI if 2.3% of the axonal fibers have MASxSR of 7.5 s^−1^ and above. The average and standard deviation of the damaged volume fraction of the whole brain (DVF) predicted using each of the 6 fraction-based metrics and AIV identified through histopathology analysis are illustrated in Fig. 10 for injured (*n* = 29) and non-injured (*n* = 13) groups and were significantly higher (*t* test *p* value < 0.05) for the injured group compared to the non-injured group.

## Discussion

### Axonal tract embedded pig brain FEM

In this study, an anisotropic axonal tract embedded pig head FEM was developed and utilized to examine different FE-derived tissue metrics for predicting TAI following rapid head rotations. As an improvement over the tract-oriented method used in previous TBI studies in which the brain responses were required to project onto the averaged axonal fiber orientation calculated for each element, in the axonal-tract embedded method used herein the details of axonal tracts were explicitly incorporated. Embedding axonal tractography into brain FEM is particularly beneficial for investigating axonal injury prediction as it can provide more morphologically relevant insight into the mechanical responses of each individual axonal fiber over time during the course of injury induced by rotation and/or impact simulations.

This study is the first to incorporate the detailed axonal tractography networks into an animal brain FEM using embedded element technique with the purpose of axonal injury metric development. Moreover, our FE model was validated against in–vitro experiments performed using the same system and under similar loading conditions that were used for our TBI experiments. The center and direction of rotation as well as the magnitude of applied kinematics were precisely controlled, measured, and replicated in simulations. Also, the extent and location of axonal injuries were precisely quantified through histopathology examinations for animal TBI data used in this study.

### Traumatic diffuse axonal injury prediction performances of FE-derived metrics

After the development of our axonal tract embedded pig FEM and validation of its deformation response, the model was used to develop injury risk curves and predict subject-sustained TAI with FE-derived metrics.

The conservative and rigorous approach used in the injury metric development and validation indicated that any of the 12 selected metrics were capable of predicting the presence of TAI with an overall accuracy rate of 73–90% and sensitivity of 75–93%. Evaluating the metrics on the same training datasets used for metrics development, which is a common approach in the literature, showed up to 8% higher prediction accuracy rates. Interestingly, for each of the fraction-based metric, the optimal cutoff value, obtained based on ROC analysis to maximize the overall prediction accuracy rate (PAR_ROC_) and AUROCs (Fig. 5), was the same or very close to the optimal ROC threshold value determined based on its correlated magnitude-based (95th percentile maximum value) metric. This similarity reassured the threshold values determined for the 6 selected magnitude-based metrics (MPS, MAS, MPSR, MASR, MPSxSR, and MASxSR) in this study. The prediction performances of fraction-based metrics were similar to their rival magnitude-based metrics; however, each has its own advantage. Magnitude-based metrics, especially axonal related ones, have potential to provide insights into the distribution of possible axonal damages areas, while fraction-based metrics, again especially axonal related one, can give an estimation of the extent/volume of axonal injury.

Slightly better predictions were found for the axonal-based metrics than metrics associated with brain tissue response in identifying the absence or presence of TAI (Tables 5, 6, 7). There are only a few computational studies in the literature that evaluated and compared the axonal injury prediction performances of the MAS and MPS metrics (Giordano and Kleiven 2014b; Sahoo et al. 2016; Sullivan et al. 2015) using a tract-oriented approach instead of an axonal embedded FE models, and similar to our findings, they found that maximum axonal tract-oriented strain (similar to MAS in this study) performed better in predicting TAI in piglets (Sullivan et al. 2015) or concussion and/or diffuse axonal injury in human (Giordano and Kleiven 2014b; Zhao et al. 2017) compared to MPS.

On the other hand, the DVF predicted by each of the FE-derived metrics was higher than the axonal injury volume obtained from histopathology analysis (Fig. 8). DVFs predicted based on the axonal-related metrics, especially AF-MASxSR_7.5_, were smaller than DVFs predicted based the brain-related metrics suggesting that incorporating axonal bundle into the FEM may have improved the overprediction of the FE-derived brain-related metrics.

Quantitative analysis of TAI prediction capability, evaluated by different prediction/discriminatory performance criteria including PAR_ROC_, PAR_50%-BLR_, PAR_50%-Weibull_, AUROC, AIC_Weibull_, and BLR *R*-squared (Tables 5, 6, 7), revealed that the metrics related to strain rate and the product of strain and strain rate were better predictors of TAI than the parameters that are solely based on strain. Only a few computational studies have investigated the metrics related to strain rate for prediction of axonal injury (King et al. 2003; Sahoo et al. 2016; Sullivan et al. 2015) and most of them agree with our findings. King et al. (2003) reconstructed the 53 subject NFL dataset and showed that strain rate and the product of strain and strain rate in the midbrain region were the best injury predictors for concussion in human. Sullivan et al. (2015) also showed that tract-oriented strain rate and strain times strain rate were better predictors of TAI than metrics solely based on strain. Only one study found maximum tract-oriented strain to be a better predictor of TAI than maximum tract-oriented strain rate (Sahoo et al. 2016).

Many in–vitro (Bar-Kochba et al. 2016; Cullen et al. 2007; Nakadate et al. 2017), in–vivo (Singh et al. 2009) and ex–vivo (Shi and Whitebone 2006) studies, that examined combinations of different strain and strain-rate magnitudes, also suggested that the extent of neuronal and axonal injury were not solely dependent on strain but also highly sensitive to strain rate. For instance, Nakadate et al. (2017) performed stretching experiments on cultured neurons to strain of 0.1, 0.15, 0.2 at strain rates of 10, 30, 50 and 70 s^−1^ and found that axonal tolerance as evidenced by the formation of axonal swellings and bulbs was strongly strain rate dependent at higher strain (0.15 and 0.2) but not under low strain (0.1). Similarly, Cullen et al. (2007) performed shear deformation (strain of 0.5) on in–vitro 3-D neuronal–astrocytic co-cultures at strain rates of 1, 10, and 30 s^−1^ and found that neuronal viability reduced significantly at the highest strain rate (30 s^−1^) and neuronal cell death increased significantly at strain rates of 30 s^−1^ and 10 s^−1^ (Cullen et al. 2007), suggesting that the extent of neuronal injury was significantly dependent on strain rate for the case of large magnitude deformation (0.5). In another in–vitro study, Bar-Kochba et al. (2016) found that at a strain of 0.3, the extent of injury as assessed by the neuronal cell death and neurite blebbing formation is higher at strain rate of 75 s^−1^ than at strain rate of 10 s^−1^ (Bar-Kochba et al. 2016). Similarly, an ex–vivo tensile stretch study of spinal cord by Shi and Whitebone (2006) also showed that strain of 0.25 at the high rates (355–519 s^−1^) caused more axonal damage, both structurally and functionally, than very low strain rates (0.006–0.008 s^−1^) even at much higher strain (up to 1). Singh et al. (2009), an in–vivo tensile stretch study of spinal nerve roots, also found that the extent of morphological/structural and functional traumatic axonal injuries are dependent on both strain and strain rate, and axons are more vulnerable at higher strain rates. Although all these experimental studies emphasized the importance of both strain and strain rate on axonal injury and suggested that higher strain rate reduces the strain threshold for axonal injury, most of computational TBI studies focus solely on strain-based metrics. The results of the current study also stress the importance of including strain-rate-based metrics (SR and SxSR) and using the combination of strain and strain rate for axonal injury prediction in the future TBI studies.

### Traumatic diffuse axonal injury thresholds

In this study, the axial logarithmic axonal bundle deformation tolerance for TAI was found to be 0.12-0.13. This predicted MAS threshold was in good agreement with injury thresholds observed in many in–vivo, in–vitro, and ex–vivo studies of isolated axons and nerve fibers. For instance, an in–vivo stretch study by Kwan et al. (1992) found axial strain of 0.12 to be the threshold for a complete conduction block in peripheral nerves. Another in–vivo study by Singh et al. (2009) also found strains of 0.16, 0.10, and 0.09, at rate of 0.01 mm/s, 1 mm/s, and 15 mm/s, respectively, as the thresholds for 50% probability of complete conduction block in the spinal nerve roots causing functional axonal injuries which agree well with the MAS threshold determined in this study. In another in–vivo axonal stretch study conducted on guinea pig optic nerve, Bain and Meaney (2000) found the ROC optimal Lagrangian strain thresholds of 0.18 for occurrence of electrophysiological impairment and 0.21 for occurrence of morphological injury as evidenced by axonal swelling or retraction bulbs detected with NF68 immunohistochemical staining (Bain and Meaney 2000). This higher threshold found in their study in comparison with our results may be attributed to three factors. First, they reported Lagrangian strain while we are reporting logarithmic strain. The equivalent logarithmic strain values for Lagrangian strain values of 0.18 and 0.21 are 0.15 and 0.17, respectively. Second, they noted that axons in the guinea pig optic nerve are undulated which might have potentially increased the strain necessary to produce injury (Bain and Meaney 2000). Third, they used NF68 immunohistochemical staining which might be less sensitive (Ibrahim et al. 2010b) to axonal damage than β-APP staining that we used in this study. β-APP has shown to be able to detect axonal flow disruption which can be occurred even before axonal structural damage that is detectable with NF68 staining (Ibrahim et al. 2010b). The MASR of 66–70 s^−1^ and MASxSR 5-7.5 s^−1^ (Tables 5, 6, 7, Fig. 5e, f) were also determined as the axial axonal bundle strain rate (SR) and strain/strain-rate combination (SxSR) tolerances for TAI. There is no experimental study currently available in the literature that examined sufficient combinations of strain and strain rate to measure SR/SxSR TAI threshold; however, many in–vitro studies (Bar-Kochba et al. 2016; Cullen et al. 2007; Nakadate et al. 2017) reported neuronal cell death, neurite/axonal swelling or bulb formations at strain rate ranging from 10 to 75 s^−1^ at different strain levels. For instance, Nakadate et al. (2017) found that formation of axonal swelling and/or bulb, similar to pathology seen in TAI, significantly increased at SR of 50 s^−1^ and 70 s^−1^ (but not lower SR) for strain of 0.15 (SxSR = 7.5–10.5) and at SR of 30 s^−1^ and above for strain of 0.2 (SxSR = 6) when compared with sham control. Although the FE-derived thresholds determined in this study compared favorably to the tissue injury thresholds found from in–vitro studies, it should be acknowledged that the scale of these measurements may not necessarily be the same as our values obtained from the meso-/macroscale FEM, while the in–vivo/in–vitro threshold values determined at the micro-/mesoscale levels. Also, the strain rates may be calculated with different processing methods and/or time frequencies among these experimental studies and ours.

The MAS thresholds determined in this study for TAI in piglets are also comparable with maximum tract-oriented strain (equivalent to MAS in this study) thresholds of 0.073-0.146 found for human concussion or diffuse axonal injury in previous TBI computational studies (Giordano and Kleiven 2014b; Sahoo et al. 2016; Zhao et al. 2017). To our knowledge, there is only one human TBI computational study (Sahoo et al. 2016) that reported maximum axonal strain-rate threshold for human DAI and their threshold (80 s^−1^) is comparable to the MASR threshold (66–70 s^−1^) that was found for TAI in piglets. Although these similarities between the TBI thresholds determined using different FEMs are encouraging, any translation or application of these thresholds to other FEM studies should be handled with caution because the FEM results are dependent on various factors such as modeling technique, material models and properties, element formation, and the model response validation process. For the strain rates, the results are also dependent on the output time frequencies and post-processing approaches.

A fundamental assumption in most of TBI studies is that the CNS tissue injury tolerances do not vary significantly in different species as evidenced by similarities in cellular structures and pathophysiology alternations of CNS tissues across species (Bain and Meaney 2000). The similarities between the deformation-related thresholds determined for TAI in piglet in this study and the thresholds reported for concussion and/or diffuse axonal injury in human in the literature support this hypothesis. Therefore, the thresholds for TAI in piglets determined herein may be translatable to human.

For the global brain tissue response, we obtained MPS of 0.29–0.30, MPSR of 120–146 s^−1^, and MPSxSR of 24.91–29.31 s^−1^ as the tolerances for TAI. These thresholds are much higher than their axonal-related corresponding thresholds (S, SR, SxSR), indicating that the maximum principal strain directions of brain tissue are not often aligned with the axonal bundles. Similar results were found in previous experimental and computational studies (Giordano and Kleiven 2014b; Sullivan et al. 2015; Tamura et al. 2007). For instance, Tamura et al. (2007) in a uniaxial stretch study on the fresh porcine brain found much smaller (~ 1/3) neuronal fiber strain, which closely correlated with strain in the neuronal fiber direction, than the global brain tissue strain. In a computational study, Sullivan et al. (2015) also reported smaller tract-oriented strain, strain rate, and SxSR thresholds than the ones for the brain tissue for predicting TAI in pigs. In another computational study, Giordano and Kleiven (2014b) also found the tract-oriented strains to be on average 75% smaller than the brain tissue principal strains.

### Limitations and path forward

Several limitations with the study should be acknowledged which are deserved for future considerations. The element embedding method used in this study did not allow for relative movement of the axonal fibers elements within the solid brain elements. While it is possible to include a slip response for the embedded elements, it is not known whether this would be an improvement in the biofidelity of the model. Also, the metric thresholds derived in this study determined the overall absence or presence of TAI based on the response of the whole brain but not the brain regional responses. The pig FEM used in this study was an idealized model in which only a few anatomical regions (corpus callosum, white matter, lateral ventricle) were segmented. Future studies should focus on investigating regional dependency of tissue tolerances and deriving region-specific metric thresholds. Also, the relatively coarse mesh of the brain FEM used in this study might have contributed to the higher DVF estimations obtained from the brain-related metrics herein. Moreover, the axonal injury volumes were quantified at every 3 mm throughout the brain in this study via histopathology analysis. Finer brain slice increments can increase the resolution of AIV calculations and thus improve the TAI assessment in future studies.

## Summary

Although diffuse axonal injury is widely recognized as one of the pathological hallmark of mTBI, a complete understanding of tissue thresholds leading to TAI is challenging. These challenges are further compounded by biofidelity limitations that exist in finite element modeling considering the anisotropy of brain tissue due to the structural distribution of axons. To address these challenges, in this study an anisotropic piglet multi-scale brain finite element model with embedded axon tractorgraphy was developed, validated, and used for predicting axonal injury during rapid head rotations. Injury thresholds derived from this model compared well with in–vitro and in–vivo studies investigating the mechanical tolerance of axonal/neuronal tissue. Predicted injury thresholds that were based on axonal response showed slightly improved TAI prediction performance with 1–7% higher prediction accuracy rate than metrics that were based on general brain tissue response. Metrics related to the product of strain and strain rate were found to be better predictors of TAI with 11–15% higher PAR than metrics that were solely based on strain. In addition, the axonal-related thresholds, especially AF-MASxSR_7.5_, provided more realistic damaged volume fraction estimations (DVF = 0.05–10.2%, for AF-MASxSR_7.5_: DVF = 0.05–4.5%), closer to the actual axonal injury volume obtained from histopathology (0.02–1.6%), than the general brain tissue thresholds (DVF = 0.11–41.2%). Overall, metrics related to the product of strain and strain rate (SxSR) were found to be much better predictors of TAI than metrics that were solely based on strain. As such, the axonal injury thresholds based on the product of strain and strain rate should be considered in the future. The FE-derived tissue injury thresholds for TAI in piglets determined herein may be directly translatable to human. In addition, the modeling methodology used in this study can be used for modeling of TBI in humans and is expected to improve the TAI prediction capability of FEM results in humans.
